# The rising tide of dementia deaths: triangulation of data from three routine data sources using the Clinical Practice Research Datalink

**DOI:** 10.1186/s12877-021-02306-7

**Published:** 2021-06-21

**Authors:** Shaleen Ahmad, Iain M Carey, Tess Harris, Derek G Cook, Stephen DeWilde, David P Strachan

**Affiliations:** Population Health Research Institute, St George’s University of London, Cranmer Terrace, SW17 0RE London, United Kingdom

**Keywords:** Dementia, Mortality, Death certificates, Cause of death, Primary care

## Abstract

**Background:**

Dementia is currently the leading certified underlying cause of death in England. We assess how dementia recording on Office for National Statistics death certificates (ONS) corresponded to recording in general practice records (GP) and Hospital Episode Statistics (HES).

**Methods:**

Retrospective study of deaths (2001-15) in 153 English General Practices contributing to the Clinical Practice Research Datalink, with linked ONS and HES records.

**Results:**

Of 207,068 total deaths from any cause, 19,627 mentioned dementia on the death certificate with 10,253 as underlying cause; steady increases occurred from 2001 to 2015 (any mention 5.3 to 15.4 %, underlying cause 2.7 to 10 %). Including all data sources, recording of any dementia increased from 13.2 to 28.6 %. In 2015, only 53.8 % of people dying with dementia had dementia recorded on their death certificates. Among deaths mentioning dementia on the death certificate, the recording of a prior diagnosis of dementia in GP and HES rose markedly over the same period. In 2001, only 76.3 % had a prior diagnosis in GP and/or HES records; by 2015 this had risen to 95.7 %. However, over the same period the percentage of all deaths with dementia recorded in GP or HES but not mentioned on the death certificate rose from 7.9 to 13.3 %.

**Conclusions:**

Dementia recording in all data sources increased between 2001 and 2015. By 2015 the vast majority of deaths mentioning dementia had supporting evidence in primary and/or secondary care. However, death certificates were still providing an inadequate picture of the number of people dying with dementia.

**Supplementary Information:**

The online version contains supplementary material available at 10.1186/s12877-021-02306-7.

## Background

Dementia is defined as the progressive, irreversible loss of cognitive functioning, usually occurring after age 65 years and encompassing many different subtypes, of which Alzheimer’s is the commonest [1]. Although no gold standard exists for dementia ascertainment [2] an estimated 850,000 people in the UK are living with dementia [1], a significant burden for health and social care systems.

Dementia is now the most commonly certified underlying cause of death in England according to Office for National Statistics (ONS) figures, accounting for 12.7 % of all registered deaths in 2017 [3]. It has overtaken cardiovascular causes as the leading underlying cause of death in the UK. ONS data suggests that death rates from dementia are steadily increasing in all older age groups (> 65 years) and in both males and females, but studies suggest that the age-specific incidence rates of dementia have fallen over the last two decades, driven primarily by a reduction of dementia diagnoses in men [4].

There has been increased public awareness of dementia due to Government initiatives such as the National Dementia Strategy [5] and the Prime Minister’s Challenge on Dementia 2020 [6]. This was associated with incentivising primary care to record dementia diagnosis in the Quality and Outcomes Framework (QOF) [7], and dementia-related Read code changes introduced after 2006 [8]. There were high national general practice participation rates in the dementia care incentive schemes of Directed Enhanced Services 18 (DES18) and Dementia Identification Scheme (DIS) of 98.5 and 76 % respectively [9]. In 2012 the Department of Health also introduced incentives to increase dementia diagnosis in secondary care, by case finding in older inpatients [8].

The aim of this work is to assess how well dementia recording in death certification relates to both routine recording in electronic general practice records and Hospital Episode Statistics (HES) over the period 2001 to 2015. The study period was chosen as it coincided with the introduction of the latest version of the International Classification of Diseases 10th revision (ICD-10) [10], so codes relating to dementia were consistent over time and in addition to showing increases in dementia death rates, it also included key policy changes to QOF [8] and hospital recording [11].

## Methods

This study used data from the Clinical Practice Research Datalink (CPRD GOLD), a national validated database of patient records collected during routine general practice consultations. Diagnoses are recorded on the system using a hierarchical clinical classification system called Read codes [12], either by staff at the time or from other sources such as discharge data from hospitals. As of 2015, CPRD had about 700 practices nationally which had contributed data (~ 7 % of the UK population), and the registered patients have been shown to be broadly representative of the UK population terms of age, sex and ethnicity [13]. The pseudo-anonymised information from this primary care database is linked to external data (HES and ONS) via an independent trusted third-party [14]. HES is a data source recording every NHS hospital admission in England containing information on clinical diagnoses [15]. ONS data include details from the death certificate for both the underlying cause of death and all causes mentioned on the death certificate. ONS death certificate data were linked to GP records via a unique patient identification number using a linkage algorithm while HES data were linked in a series of deterministic linkage steps [14].

### Study Sample

In order that findings reflect trends over time, rather than differences due to practices entering and exiting CPRD over time, we restricted our study to 153 English practices that contributed up-to-standard data over the entire study period 2001 to 2015. One of the criteria for this quality control measure carried out by CPRD is number of recorded deaths within a practice [16]. Additionally, the study was restricted to English practices as patients residing outside England are not linked to ONS mortality data or HES within CPRD [13].

All deaths over the period 2001–2015 were identified from the electronic General Practice (GP) records and then linked to ONS mortality to confirm the death. There is near agreement between the two sources, with 98.2 % of deaths in ONS reported to be also identified in CPRD [17]. However, it is common for the CPRD date associated with the death to be on average 1 month later [18], so we used the ONS date for the date of death in our analyses. Codes for dementia of any type were identified within each of the GP, HES and ONS datasets. A patient was defined as having “died with dementia” if they had any evidence of a diagnosis of dementia on any of the three data sources.

### Dementia recording on GP electronic records

Within the patient’s primary care record we searched for diagnoses of dementia (“GP diagnosis”) using specific Read codes indicating a diagnosis for dementia based on a standard code list in the QOF [7] (Supplementary Table 1). Additionally, we defined two further groups based on codes associated with dementia: “GP awareness”, which is related to suspected memory impairment or other codes indicative of dementia without a formal diagnosis of dementia (Supplementary Table 2); “GP administration”, which refers to other administrative codes on the GP system relating to dementia (Supplementary Table 3). “Any GP record” of dementia was based on the “GP diagnosis” category only, while the other categories were used to investigate recording patterns in patients identified as having dementia from their death certificate. To check whether length of registration prior to death had influenced recording, we carried out sensitivity analyses restricted to patients registered in their practice for at least a year before death.

### Dementia recording on HES and ONS

Within the linked hospitalisations and mortality datasets we searched all records for evidence of a diagnosis of dementia, which are both coded using International Classification of Diseases version 10 (ICD-10) codes (Supplementary Table 4). For hospitalisations, HES Admitted Patient Care (HES APC) data are collected on all admissions to National Health Service (NHS) hospitals in England [15], with the primary reason for admission coded along with other major co-morbidities. Any recording of dementia was counted as evidence of a dementia diagnosis. For mortality, the ONS dataset extracts summary information from the Medical Certificate of the Cause of Death (MCCD), the document completed by a doctor involved in the care of the patient. It has two parts: conditions leading to death are recorded in Part 1 and other significant conditions contributing to the death are recorded in Part 2 [19]. Any recording was counted as evidence of a dementia diagnosis, but we sometimes make the distinction between “listed as underlying cause” (Part 1 only) and “any mention” (Part 1 or 2).

### Statistical Analyses

Prevalence estimates of dementia recording are reported in the text with 95 % confidence intervals calculated from the proportion recorded. A visual summary of the overlap recorded in the three data sources (GP, HES records and ONS mortality statistics) was represented by Venn diagrams in 2001 and 2015, using approximate scaling for each to represent the percentage of deaths recorded in the three data sources. A fixed-sized rectangle borders the diagram and represents 100 % of deaths in each year. The plots were carried out using the Venn Diagram Plotter freely available from the Pacific Northwest National Laboratory [20].

## Results

### Trends among all deaths

Table 1 and Supplementary Fig. 1 summarise the trends in annual dementia recording in death certification, GP records and HES data in all deaths in the study population between 2001 and 2015. There were a total of 207,068 deaths in the study population, of which 19,627 (9.5 %) deaths had any mention of dementia (either as a contributory or underlying cause of death) on the death certificate. Dementia was listed as the underlying cause in 10,253 (5.0 %) deaths. There was an increasing trend in dementia recording in records from all three data sources: death certification, electronic GP health records and HES data.

**Table 1:** Summary of dementia recording among all deaths from 2001–2015 (*n* = 207,068)

**Year of Death**	**Total Deaths**	**Dementia recording on Death Certificate**					**Prior Dementia diagnosis in GP or HES records**							
		**Any mention**		**Listed as underlying cause only**			**GP diagnosis**		**HES diagnosis**		**GP or HES diagnosis plus death certificate**		**GP or HES diagnosis but not on death certificate**	
	**n**	**n**	**% of total**	**n**	**% of total **	**% of dementia deaths only**^a^	**n**	**% of total**	**n**	**% of total **	**n**	**% of total **	**n**	**% of total **
2001	13,796	733	5.3 %	376	2.7 %	51.3 %	1,077	7.8 %	1,079	7.8 %	1,651	12.0 %	1,092	7.9 %
2002	14,099	820	5.8 %	387	2.7 %	47.2 %	1,242	8.8 %	1,322	9.4 %	1,898	13.5 %	1,280	9.1 %
2003	14,386	919	6.4 %	403	2.8 %	43.9 %	1,375	9.6 %	1,470	10.2 %	2,105	14.6 %	1,358	9.4 %
2004	13,997	927	6.6 %	398	2.8 %	42.9 %	1,367	9.8 %	1,515	10.8 %	2,090	14.9 %	1,316	9.4 %
2005	13,863	968	7.0 %	419	3.0 %	43.3 %	1,399	10.1 %	1,635	11.8 %	2,130	15.4 %	1,299	9.4 %
2006	13,790	1,014	7.4 %	414	3.0 %	40.8 %	1,420	10.3 %	1,705	12.4 %	2,196	15.9 %	1,309	9.5 %
2007	13,753	1,087	7.9 %	451	3.3 %	41.5 %	1,602	11.6 %	1,886	13.7 %	2,361	17.2 %	1,386	10.1 %
2008	14,163	1,287	9.1 %	525	3.7 %	40.8 %	1,802	12.7 %	2,169	15.3 %	2,653	18.7 %	1,507	10.6 %
2009	13,562	1,374	10.1 %	547	4.0 %	39.8 %	1,768	13.0 %	2,276	16.8 %	2,710	20.0 %	1,472	10.9 %
2010	13,921	1,478	10.6 %	580	4.2 %	39.2 %	1,859	13.4 %	2,486	17.9 %	2,899	20.8 %	1,562	11.2 %
2011	13,607	1,565	11.5 %	988	7.3 %	63.1 %	1,931	14.2 %	2,643	19.4 %	3,035	22.3 %	1,588	11.7 %
2012	14,058	1,823	13.0 %	1,149	8.2 %	63.0 %	2,150	15.3 %	2,954	21.0 %	3,369	24.0 %	1,675	11.9 %
2013	14,274	1,868	13.1 %	1,160	8.1 %	62.1 %	2,307	16.2 %	3,026	21.2 %	3,469	24.3 %	1,716	12.0 %
2014	13,718	1,909	13.9 %	1,250	9.1 %	65.5 %	2,394	17.5 %	3,075	22.4 %	3,665	26.7 %	1,756	12.8 %
2015	12,081	1,855	15.4 %	1,206	10.0 %	65.0 %	2,562	21.2 %	2,870	23.8 %	3,381	28.0 %	1,605	13.3 %
All	207,068	19,627	9.5 %	10,253	5.0 %	52.2 %	26,255	12.7 %	32,111	15.5 %	39,612	19.1 %	21,921	10.6 %

^a^ Denominator here is all deaths with any mention of dementia on death certificate

In the ONS data any mention of dementia on the death certificate over the study period tripled, from 5.3 % (95%CI 4.9 - 5.7 %) to 15.4 % (95%CI 14.7 - 16.0 %) whilst recording of dementia as the underlying cause of death increased from 2.7 % (95%CI 2.5 - 3.0 %) to 10.0 % (95%CI 9.4 - 10.6 %). Of deaths recorded as dementia on the death certificate, the proportion recording it as the underlying cause increased over this period from 51.3 % (95%CI 50.5 - 52.1 %) to 65.0 % (95%CI 64.2 - 65.9 %). GP recording of dementia on electronic GP health records increased from 7.8 % (95%CI 7.4 - 8.3 %) of all deaths in 2001 to 21.2 % (95%CI 20.5 - 21.9 %) in 2015. HES records showed a similar pattern of increase in coding of dementia over the study period, with 7.8 % (95%CI 7.4 - 8.3 %) of records including a dementia code in 2001 increasing to 23.8 % (95%CI 23.0 - 24.5 %) in 2015. When we limited analyses to patients registered in their practice for at least a year, findings were similar (Supplementary Table 5).

**Fig. 1:**
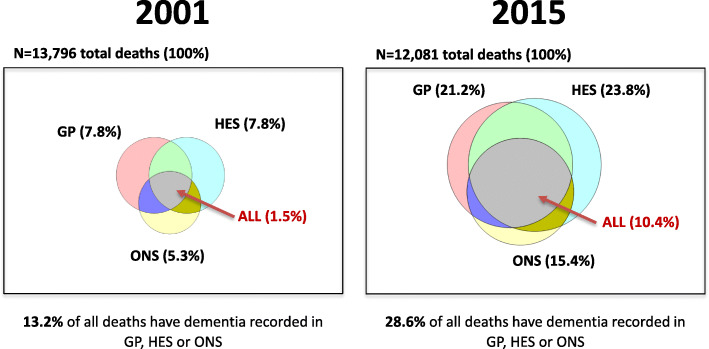
Triangulation of GP, HES and ONS recording of dementia in 2015 vs. 2001 for all deaths. Footnote: All percentages are for deaths from any cause indicated by; (i) for GP (diagnostic codes), (ii) for HES (diagnostic codes) and (iii) for ONS (any mention of dementia on death certificate). Approximate scaling for each circle has been used to represent the relative contribution of each of the data sources.

The triangulation of dementia recording from all 3 sources is shown in Fig. 1 for 2001 and 2015. There was an increase in dementia recorded in any one of the data sources, from 13.2 % (95%CI 12.7 - 13.8 %) of all deaths with dementia recorded in 2001 to 28.6 % (95%CI 27.8 - 29.4 %) of all deaths in 2015. In particular there was a substantial increase in subjects with a dementia diagnosis from all 3 sources (grey area) rising from 1.5 % (95%CI 1.3 - 1.7 %) in 2001 to 10.4 % (95%CI 9.8 - 10.9 %) in 2015.

However, relying on the death certificate alone for a diagnosis of dementia gives an incomplete picture of the number of people in England dying with dementia (i.e. all patients who die with a diagnosis of dementia from any source). While the percentage of all deaths with a diagnosis recorded in any of GP, HES or ONS captured by death certificates has risen from 40.2 % to 2001 (5.3 %/13.2 %), it is still only approximately half (53.8 % = 15.4 %/28.6 %) of these deaths in 2015 (Fig. 1). Due to the increase in GP and HES recording, this means the percentage of all deaths with a dementia diagnosis recorded in GP or HES but not mentioned on the death certificate, has risen from 7.9 to 13.3 %.

### Trends among deaths with any mention of dementia on the death certificate

**Fig. 2:**
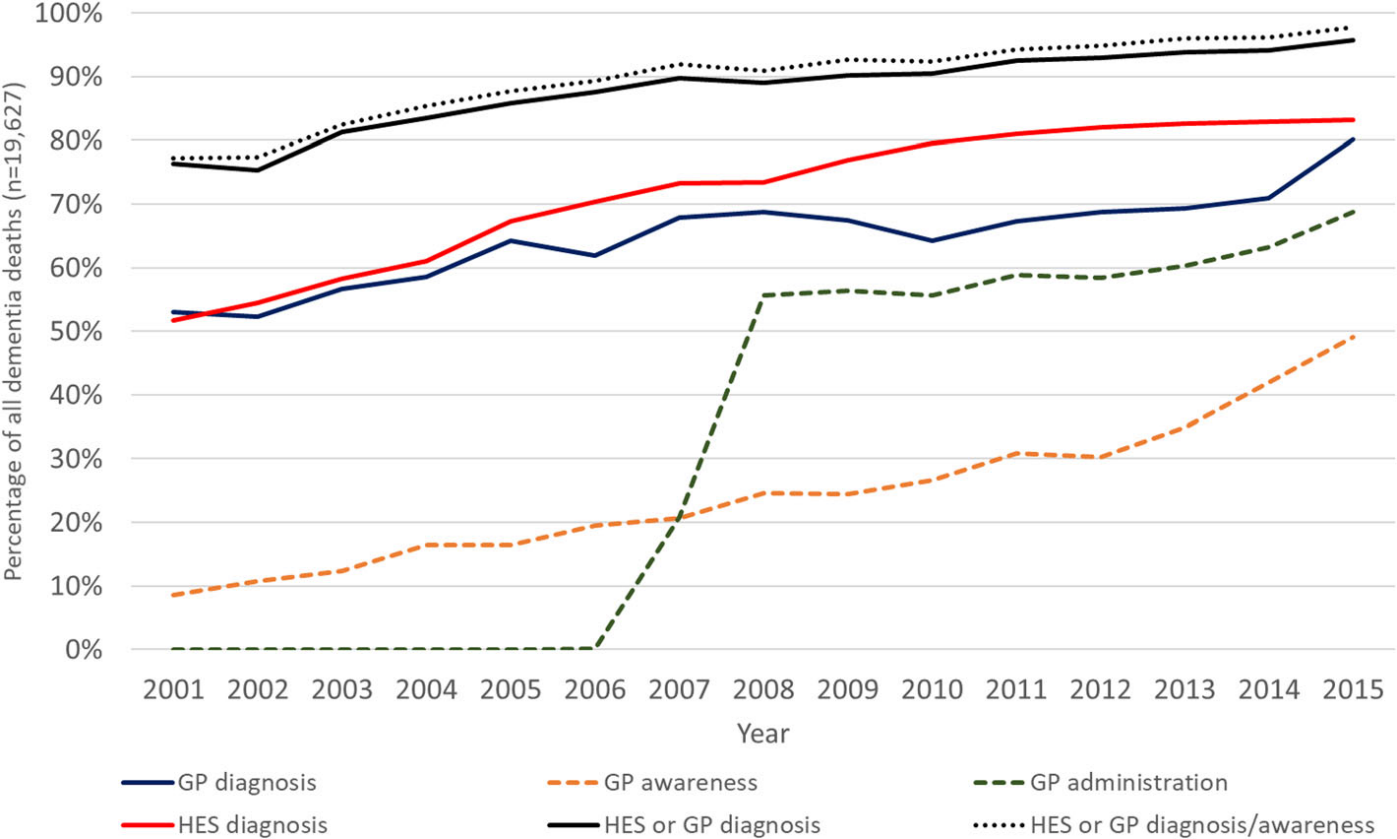
Trends in the recording of dementia diagnoses and other information in GP and HES among all death certifications with mention of dementia 2001–2015 (*n* = 19,627). Footnote: Definition of categories was as follows: GP diagnosis = includes specific Read codes indicating a diagnosis for dementia, GP awareness = includes Read codes related to suspected memory impairment or other codes indicative of Dementia, GP administration = Other administrative codes on GP system relating to dementia, Any GP recording = Any of GP diagnosis, awareness or administration, HES diagnosis = refers to admission records which mention dementia either as primary cause of admission or other

Figure 2 illustrates trends in dementia related recording in GP or HES records amongst patients with any mention of dementia on the death certificate (Supplementary Table 6 provides the underlying data). The proportion of patients who had a GP (red line) or HES (blue line) diagnosis shows a similar pattern of increase over this period. Together, this represents an increase from 76.3 to 95.7 % in the percentage who had a prior diagnosis in GP and/or HES records. In addition, trends in codes indicating GP dementia awareness showed a steady increase over this period, while the use of GP administrative codes quickly increased once they were introduced with QOF circa 2006. Putting all these codes together, the percentage of deaths with dementia mentioned on the death certificate that also had supporting evidence in either GP and/or HES records rose from 77.2 % to 2001 to 97.7 % in 2015.

## Discussion

### Summary of main findings

There is increased recording of dementia diagnoses across both primary and secondary care between 2001 and 2015, and this is reflected in an increased number of deaths recording dementia, both as a contributory or underlying cause, a trend also seen in national data [3]. By 2015, the vast majority (97.7 %) of deaths with dementia mentioned on the death certificate had existing evidence of dementia recorded in primary and/or secondary care records. However, over the same period, the percentage of people dying with dementia, but not of dementia has substantially increased. In particular, while the percentage of deaths with dementia mentioned on the death certificate, but not as underlying cause, has increased, this has not kept pace with the rise in dementia diagnoses in primary and secondary care records. Thus by 2015 only half of those dying with dementia have it mentioned on the death certificate as a contributory cause. While not surprising, it does mean that death certification provides an inadequate picture of the prevalence of dementia at the time of death.

### Strengths and limitations

One of the major strengths of this study is the breadth of geographical coverage and basis in an unselected population from a large, validated longitudinal primary care database that is broadly representative of the population [13]. The time period assessed includes key dementia related policy changes to QOF [8] and in contrast to some other studies, we have restricted to practices that contributed data over the entire study period for consistency.

In addition to the comprehensive data linkage between the databases [13], HES has universal coverage in England, which is a key strength [15], although due to variation in quality of coding, data about co-morbidities such as dementia may be entered inconsistently across sites. As well as issues with missing data HES does not include outpatient data so any patients with dementia who attended a dementia/memory assessment clinic will not have been coded in HES, though diagnoses from such letters should have been coded in primary care records and therefore included in CPRD. HES-CPRD data first became available from April 1997 [15], which may impact on the results as deaths from earlier in the study period would have a maximum of three years of prior hospital data. The time period assessed includes policy change related to hospital recording of dementia introduced in 2012 [11].

We were constrained to using data for England only, as CPRD linkage to HES and ONS is limited to this area [14]. Data quality in CPRD is reliant on accurate coding by primary care staff and there may be variability in coding across sites, as well as issues with the Read codes associated with dementia as coding changes were introduced during the study period. This is illustrated by the sharp increase in recorded GP dementia awareness after 2006 which corresponded with the introduction of new dementia related codes incentivising QOF after 2006 [7, 8]. There was also a coding change affecting ONS records in 2010, with a modification in the rules on death certification, aimed at improving the accuracy of recording of the underlying cause of death [21]. Whilst this did not impact greatly on the all-cause dementia rates (Supplementary Fig. 2), it has affected the subtypes of dementia recorded, as a correction was made to the coding of vascular dementia and previously coded cerebrovascular disease was corrected to vascular dementia [22]. As a result of these coding changes there may have been some changes in the proportions of underlying cause within the any-mention of dementia group at the 2010 to 2011 transition. After this coding change the number of deaths with any mention of dementia on the death certificate continued to rise.

### Comparison with other studies

A recent systematic review [23] of the validity of dementia diagnoses in routinely collected UK electronic health records reported generally high validity, although all studies included were at significant risk of bias. Individual studies have also previously addressed recording of dementia across different data sources. Brown et al. found higher levels of agreement between GP and HES data (95 %) than we reported, but in a small sample of women only (*n* = 340) [24]. Perera et al. linked dementia diagnoses from a London mental health database to ONS mortality data from 2006 to 2013 and reported an increase in dementia recording over this period, consistent with our findings [25]. A much larger study (47,386 people with dementia) examined the diagnostic validity of dementia in CPRD records from 1998 to 2010, also using HES and ONS records, as we did [26]. Whilst not directly comparable to our study, as they did not examine trends over time and we provide more recent data; they found that 8 % had evidence of a dementia diagnosis across all three sources during this period, compared to our figures of 1.5 % in 2001 and 10.4 % in 2015 [26].

In UK, the Cognitive Functioning in Ageing Study (CFAS) provided important data on trends over time (1989–2016), in accuracy of death certification of dementia, from their large cohort study of patients aged > 65 years (*n* = 26,699) [4, 27]. They demonstrated an increase in overall unadjusted prevalence of dementia on death certificates (5.3 to 25.9 %) and an increase in sensitivity of dementia reporting on death certificates compared to a gold-standard of study clinical diagnosis of dementia (from 21 % in CFAS I to 45.2 % in CFAS II) [4, 27]. These are consistent with our findings, indicating a similar trend of increased recording of dementia on death certificates, but with a recognition that death certificates still give an inadequate picture of the number of people dying with dementia.

It is not generally well understood what influences decisions regarding diagnoses recorded in the death certificate [28]. During our study period we observed a steady increase in dementia appearing on death certificates between 2001 and 2015, while at the same time age-specific incidence rates of dementia were falling in the UK [27]. This trend has been replicated in other cohort studies in Europe and North America, suggesting that over the last 25 years the incidence rate may have declined by 13 % per decade [29]. It seems plausible that the improvements in the management of cardiovascular disease over time has contributed to this reduction, particularly for vascular dementia [30]. This might also result in a deferral of dementia to older age, however with people living longer, age at death will have increased over this time too, so the likelihood of having dementia by the time of death would almost certainly have risen as well. This could explain the increasing numbers of deaths from dementia observed in this study. Other potential contributing factors over the study period could include the increased public and medical awareness due to activity by campaigning groups and government, as well as increased referral access to memory clinics. The National Dementia Strategy, launched in 2009, has previously been shown to be associated with a significant increase in dementia diagnosis rates and prescriptions of antidementia drugs [31]. Dementia deaths in this study with a prior diagnosis of dementia in both electronic GP and hospital records has increased from 76.3 % to 2001 to 95.7 % in 2015 which may reflect a change in clinicians’ attitudes and less reluctance in entering a diagnosis of dementia due to concerns about stigma and increased case finding and confidence in management [32].

### Implications for practice and public health policy

Changes in QOF in primary care and in policy relating to hospital inpatients, both designed to increase the diagnosis rate of dementia, have led to a greater awareness of dementia in clinical practice and increased recording of dementia on death certificates as a primary or contributory cause. Nevertheless, only half of those dying with a prior diagnosis of dementia had dementia recorded on their death certificate in 2015. The high and increasing proportion of people recognized to be dying “with” dementia (in addition to those dying “of” dementia) has important cost and workforce implications for both health and social care service planning for care of older people.

### Further work

The introduction of Independent Medical Examiners (IME) from April 2019 [33] could impact on dementia recording rates, as the role of the IME extends to discussions with relatives before deciding on cause of death. Repeating the study in the future and comparing it to current findings may give further insights into dementia detection and recording in primary care. Previous work has demonstrated significant regional variations in dementia recording [34] this could also be investigated as a potential explanatory factor.

## Conclusions

Our study demonstrates that between 2001 and 2015 in England, dementia recording in GP electronic records, hospital records and death certification increased. By 2015 the vast majority of deaths mentioning dementia on the death certificate had prior evidence of diagnosis in primary and/or secondary care (97.7 %); a marked improvement from 2001. It emphasizes that when dementia is mentioned on death certificates there is good evidence backing this up. However, death certificates still give an inadequate picture of the total number of people dying with dementia; in 2015 only half of those people dying with dementia had dementia recorded on their death certificates.

## Supplementary Information

**Additional file 1.**

## Data Availability

The data that support the findings of this study are available from CPRD but restrictions apply to the availability of these data, which were used under license for the current study, and so are not publicly available.

## Abbreviations

CFAS: Cognitive Functioning in Ageing Study
CPRD: Clinical Practice Research Datalink
DIS: Dementia Identification Scheme
DES: Directed Enhances Services
GP: General Practice
HES: Hospital Episode Statistics
ICD: International Classification of Diseases
IME: Independent Medical Examiners
MCCD: Medical Certificate of the Cause of Death
NHS: National Health Service
ONS: Office for National Statistics
QOF: Quality and Outcomes Framework
