# Supplementary material for: The rising tide of dementia deaths: triangulation of data from three routine data sources using the Clinical Practice Research Datalink
Source: BMC Geriatr. 2021 Jun 21;21:375. doi: 10.1186/s12877-021-02306-7 (PMC8218386; doi:10.1186/s12877-021-02306-7)
Supplement: Supplementary file 1 — Additional file 1. [file 12877_2021_2306_MOESM1_ESM.docx]

**Supplementary Table 1:** Read codes indicating dementia diagnosis on GP record (“GP diagnosis”)

| **Readcode** | **Description** |
| --- | --- |
| **1461.00** | H/O: dementia |
| **3AE3.00** | GDS level 4 - moderate cognitive decline |
| **3AE4.00** | GDS level 5 - moderately severe cognitive decline |
| **3AE5.00** | GDS level 6 - severe cognitive decline |
| **3AE6.00** | GDS level 7 - very severe cognitive decline |
| **E00..00** | Senile and presenile organic psychotic conditions |
| **E00..11** | Senile dementia |
| **E00..12** | Senile/presenile dementia |
| **E000.00** | Uncomplicated senile dementia |
| **E001.00** | Presenile dementia |
| **E001000** | Uncomplicated presenile dementia |
| **E001100** | Presenile dementia with delirium |
| **E001200** | Presenile dementia with paranoia |
| **E001300** | Presenile dementia with depression |
| **E001z00** | Presenile dementia NOS |
| **E002.00** | Senile dementia with depressive or paranoid features |
| **E002000** | Senile dementia with paranoia |
| **E002100** | Senile dementia with depression |
| **E002z00** | Senile dementia with depressive or paranoid features NOS |
| **E003.00** | Senile dementia with delirium |
| **E004.00** | Arteriosclerotic dementia |
| **E004.11** | Multi infarct dementia |
| **E004000** | Uncomplicated arteriosclerotic dementia |
| **E004100** | Arteriosclerotic dementia with delirium |
| **E004200** | Arteriosclerotic dementia with paranoia |
| **E004300** | Arteriosclerotic dementia with depression |
| **E004z00** | Arteriosclerotic dementia NOS |
| **E00y.00** | Other senile and presenile organic psychoses |
| **E00y.11** | Presbyophrenic psychosis |
| **E00z.00** | Senile or presenile psychoses NOS |
| **E012.00** | Other alcoholic dementia |
| **E012.11** | Alcoholic dementia NOS |
| **E012000** | Chronic alcoholic brain syndrome |
| **E02y100** | Drug-induced dementia |
| **E041.00** | Dementia in conditions EC |
| **Eu00.00** | [X]Dementia in Alzheimer's disease |
| **Eu00000** | [X]Dementia in Alzheimer's disease with early onset |
| **Eu00011** | [X]Presenile dementia,, Alzheimer's type |
| **Eu00012** | [X]Primary degenerative dementia, Alzheimer's type, presenile onset |
| **Eu00013** | [X]Alzheimer's disease type 2 |
| **Eu00100** | [X]Dementia in Alzheimer's disease with late onset |
| **Eu00111** | [X]Alzheimer's disease type 1 |
| **Eu00112** | [X]Senile dementia,,Alzheimer's type |
| **Eu00113** | [X]Primary degenerative dementia of Alzheimer's type, senile onset |
| **Eu00200** | [X]Dementia in Alzheimer's dis, atypical or mixed type |
| **Eu00z00** | [X]Dementia in Alzheimer's disease, unspecified |
| **Eu00z11** | [X]Alzheimer's dementia unspecified |
| **Eu01.00** | [X]Vascular dementia |
| **Eu01.11** | [X]Arteriosclerotic dementia |
| **Eu01000** | [X]Vascular dementia of acute onset |
| **Eu01100** | [X]Multi-infarct dementia |
| **Eu01111** | [X]Predominantly cortical dementia |
| **Eu01200** | [X]Subcortical vascular dementia |
| **Eu01300** | [X]Mixed cortical and subcortical vascular dementia |
| **Eu01y00** | [X]Other vascular dementia |
| **Eu01z00** | [X]Vascular dementia, unspecified |
| **Eu02.00** | [X]Dementia in other diseases classified elsewhere |
| **Eu02000** | [X]Dementia in Pick's disease |
| **Eu02100** | [X]Dementia in Creutzfeldt-Jakob disease |
| **Eu02200** | [X]Dementia in Huntington's disease |
| **Eu02300** | [X]Dementia in Parkinson's disease |
| **Eu02400** | [X]Dementia in human immunodeficiency virus [HIV] disease |
| **Eu02500** | [X]Lewy body dementia |
| **Eu02y00** | [X]Dementia in other specified diseases classified elsewhere |
| **Eu02z00** | [X] Unspecified dementia |
| **Eu02z11** | [X] Presenile dementia NOS |
| **Eu02z12** | [X] Presenile psychosis NOS |
| **Eu02z13** | [X] Primary degenerative dementia NOS |
| **Eu02z14** | [X] Senile dementia NOS |
| **Eu02z15** | [X] Senile psychosis NOS |
| **Eu02z16** | [X] Senile dementia, depressed or paranoid type |
| **Eu04100** | [X]Delirium superimposed on dementia |
| **F110.00** | Alzheimer's disease |
| **F110000** | Alzheimer's disease with early onset |
| **F110100** | Alzheimer's disease with late onset |
| **F111.00** | Pick's disease |
| **F112.00** | Senile degeneration of brain |
| **F116.00** | Lewy body disease |

**Supplementary Table 2:** Read codes indicating that GP has concern regarding dementia (“GP awareness”)

| **Readcode** | **Description** |
| --- | --- |
| **14Od.00** | At risk of dementia |
| **1B1A.00** | Memory loss - amnesia |
| **1B1A.11** | Amnesia symptom |
| **1B1A.12** | Memory loss symptom |
| **1B1A.13** | Memory disturbance |
| **1B1A000** | Temporary loss of memory |
| **1B1A100** | Short-term memory loss |
| **1S23.00** | Memory impairment |
| **1JA2.00** | Suspected dementia |
| **38C1000** | Assessment for dementia |
| **3A...12** | Dementia assessment |
| **3A10.00** | Memory: own age not known |
| **3A20.00** | Memory: present time not known |
| **3A30.00** | Memory: present place not known |
| **3A40.00** | Memory: present year not known |
| **3A50.00** | Memory: own DOB not known |
| **3A60.00** | Memory: present month not known |
| **3A70.00** | Memory: important event not known |
| **3A80.00** | Memory: important .person not known |
| **3A91.00** | Memory: count down unsuccess. |
| **3AA1.00** | Memory: address recall unsuccesful |
| **3AD..00** | Dementia test |
| **3AD1.00** | Ten item dementia test |
| **3AD2.00** | Thirty seven item dementia test |
| **3AD3.00** | Six item cognitive impairment test |
| **3AE..00** | Global deterioration scale: assessment of primary degenerative dementia |
| **3AE2.00** | GDS level 3 - mild cognitive decline |
| **7P10400** | Neuropsychology test of memory |
| **8HTY.00** | Referral to memory clinic |
| **8IAe000** | Dementia advance care plan declined |
| **8IAe200** | Dementia advance care plan review declined |
| **8IBB000** | Dementia assessment not indicated |
| **8IEn.00** | Referral to memory clinic declined |
| **8T05200** | Referral for dementia assessment |
| **9Nk1.00** | Seen in memory clinic |
| **9OqD.00** | Dementia screening questionnaire sent to patient |
| **9OqE.00** | Dementia screening questionnaire completed |
| **9OqF.00** | No response to dementia screening invitation |
| **E2A1000** | Mild memory disturbance |
| **E2A1100** | Organic memory impairment |
| **R00z011** | [D]Memory deficit |
| **Z7CE400** | Memory disturbance (& amnesia (& symptom)) |
| **Z7CE412** | Memory loss symptom |
| **Z7CE413** | Memory loss - amnesia |
| **Z7CE414** | Memory disturbance |
| **Z7CE415** | Loss of memory |
| **Z7CE500** | Forgetful |
| **Z7CE600** | Amnesia |
| **Z7CE611** | Memory loss |
| **Z7CE612** | Memory gone |
| **Z7CE613** | Dysmnesia |
| **Z7CE614** | Memory loss - amnesia |
| **Z7CE615** | Loss of memory |
| **Z7CE616** | LOM - Loss of memory |
| **Z7CEA12** | Impairment of immediate recall |
| **Z7CEC11** | Loss of memory for recent events |
| **Z7CEC12** | No memory for recent events |
| **Z7CED00** | Amnesia for day to day facts |
| **Z7CEE00** | Amnesia for important personal information |
| **Z7CEH00** | Memory impairment |
| **Z7CEH11** | Memory dysfunction |
| **Z7CEH12** | Memory deficit |
| **Z7CEH14** | Memory problem |
| **Z7CEH15** | Poor memory |
| **Z7CEJ00** | Memory lapses |
| **Z7CEK00** | Minor memory lapses |
| **Z7CEL00** | Mild memory disturbance |
| **Z7CEN00** | Confabulation |
| **Z7CEN11** | Invents experiences to compensate for loss of memory |
| **Z7CEO00** | Momentary confabulation |
| **Z7CEP00** | Fantastical confabulation |
| **Z7CF.00** | Observations of memory performance |
| **Z7CF100** | Memory recall normal |
| **Z7CF111** | Global memory recall within normal limits |
| **Z7CF300** | Immediate recall observations |
| **Z7CF400** | Digit span performance |
| **Z7CF500** | Digit span forwards |
| **Z7CF700** | Short-term memory within normal limits |
| **Z7CF711** | Good short-term memory |
| **Z7CF712** | No problem with short-term memory |
| **Z7CF800** | Poor short-term memory |
| **Z7CF811** | Short-term memory loss |
| **Z7CF900** | Able to recall random address at five minutes |
| **Z7CFA00** | Unable to recall random address at five minutes |
| **Z7CFC00** | Unable to recall five digit number at five minutes |
| **Z7CFF00** | Forgets what was going to do |
| **Z7CFG00** | Forgets what was going to say |
| **Z7CFH00** | Forgets recent activities |
| **Z7CFI00** | Forgets what has just done |
| **Z7CFJ00** | Forgets what has just said |
| **Z7CFK00** | Forgets what has just read |
| **Z7CFL00** | Forgets what has just seen |
| **Z7CFM00** | Forgets what has just heard |
| **Z7CFO00** | Poor long-term memory |
| **Z7CFO11** | Long-term memory loss |
| **Z7CFQ00** | Unable to remember own date of birth |
| **Z7CFS00** | Unable to remember own age |
| **Z7CFS11** | Cannot remember own age |
| **Z7CFU00** | Unable to remember day of the week |
| **Z7CFW00** | Unable to remember today's date |
| **Z7CFa00** | Unable to remember current year |
| **Z7CFe00** | Unable to remember name of current prime minister |
| **Z7CFg00** | Cannot remember names of intimates |
| **Z7CFh00** | Cannot remember birth dates of children |
| **Z7CFi00** | Cannot remember wedding anniversary |
| **Z7CFq00** | Unable to remember motor skills |
| **Z7CFs00** | Unable to remember new motor skills |
| **Z7CFw00** | Memory aided by use of diary |
| **Z7CFx00** | Memory aided by use of labels |
| **Z7CFz00** | Memory aided by use of lists |
| **ZR3V.00** | Clinical dementia rating scale |
| **ZR3V.11** | DRS - Clinical dementia rating scale |
| **ZR3V.12** | CDR - Clinical dementia rating scale |
| **ZR3V.13** | Dementia rating scale |
| **ZRV9.00** | Kendrick battery for detection of dementia in the elderly |
| **ZRV9.11** | Kendrick cognitive tests for the elderly |

**Supplementary Table 3:** Read codes for GP related dementia administration (“GP administration”)

| **Readcode** | **Description** |
| --- | --- |
| **38C1300** | Assessment of psychotic and behavioural symptoms of dementia |
| **66h..00** | Dementia monitoring |
| **6AB..00** | Dementia annual review |
| **8BM0200** | Dementia medication review |
| **8BPa.00** | Antipsychotic drug therapy for dementia |
| **8CMZ.00** | Dementia care plan |
| **8CMZ000** | Dementia care plan agreed |
| **8CMZ100** | Dementia care plan reviewed |
| **8CMZ200** | Dementia care plan declined |
| **8Hla.00** | Referral to dementia care advisor |
| **8T05.00** | Referral to dementia service |
| **8T05000** | Referral to dementia support organisation |
| **8T05100** | Referral to dementia support organisation declined |
| **9Ou..00** | Dementia monitoring administration |
| **9Ou1.00** | Dementia monitoring first letter |
| **9Ou2.00** | Dementia monitoring second letter |
| **9Ou3.00** | Dementia monitoring third letter |
| **9Ou4.00** | Dementia monitoring verbal invite |
| **9Ou5.00** | Dementia monitoring telephone invite |
| **9hD..00** | Exception reporting: dementia quality indicators |
| **9hD0.00** | Excepted from dementia quality indicators: Patient unsuitable |
| **9hD1.00** | Excepted from dementia quality indicators: Informed dissent |

**Supplementary Table 4:** ICD-10 for dementia recording in HES and ONS datasets

| **ICD-10 Code** | **Description** |
| --- | --- |
| **F00*** | Dementia in Alzheimer’s disease |
| **F01*** | Vascular dementia |
| **F02*** | Dementia in other diseases classified elsewhere |
| **F03*** | Unspecified dementia |
| **G30*** | Alzheimer's disease |

*Note: All sub-codes included

**Supplementary Table 5:** Summary of dementia recording among all deaths from 2001-2015 (n=207,068) – One Year Registration Only

| **Year of Death** | **Total Deaths** | **Dementia recording on Death Certificate** | | | | | **Prior Dementia diagnosis in GP or HES records** | | | | | | | |
| --- | --- | --- | --- | --- | --- | --- | --- | --- | --- | --- | --- | --- | --- | --- |
|  |  | **Any mention** | | **Listed as underlying cause only** | | | **GP diagnosis** | | **HES diagnosis** | | **GP or HES diagnosis plus death certificate** | | **GP or HES diagnosis but not on death certificate** | |
|  | **n** | **n** | **% of total deaths** | **n** | **% of total deaths** | **% of dementia deaths only*** | **n** | **% of total deaths** | **n** | **% of total deaths** | **n** | **% of total deaths** | **n** | **% of total deaths** |
| 2001 | 12,311 | 277 | **2.3%** | 274 | **2.2%** | **50.3%** | 551 | **4.5%** | 863 | **7.0%** | 811 | **6.6%** | 1,281 | **10.4%** |
| 2002 | 12,567 | 306 | **2.4%** | 341 | **2.7%** | **47.3%** | 647 | **5.1%** | 1,001 | **8.0%** | 1,036 | **8.2%** | 1,512 | **12.0%** |
| 2003 | 12,779 | 302 | **2.4%** | 385 | **3.0%** | **44.0%** | 687 | **5.4%** | 1,096 | **8.6%** | 1,143 | **8.9%** | 1,654 | **12.9%** |
| 2004 | 12,320 | 289 | **2.3%** | 385 | **3.1%** | **42.9%** | 674 | **5.5%** | 1,062 | **8.6%** | 1,141 | **9.3%** | 1,592 | **12.9%** |
| 2005 | 12,296 | 313 | **2.5%** | 411 | **3.3%** | **43.2%** | 724 | **5.9%** | 1,118 | **9.1%** | 1,270 | **10.3%** | 1,679 | **13.7%** |
| 2006 | 12,185 | 307 | **2.5%** | 455 | **3.7%** | **40.3%** | 762 | **6.3%** | 1,112 | **9.1%** | 1,276 | **10.5%** | 1,670 | **13.7%** |
| 2007 | 12,190 | 348 | **2.9%** | 487 | **4.0%** | **41.7%** | 835 | **6.8%** | 1,287 | **10.6%** | 1,454 | **11.9%** | 1,856 | **15.2%** |
| 2008 | 12,473 | 414 | **3.3%** | 569 | **4.6%** | **42.1%** | 983 | **7.9%** | 1,426 | **11.4%** | 1,671 | **13.4%** | 2,059 | **16.5%** |
| 2009 | 11,925 | 412 | **3.5%** | 619 | **5.2%** | **40.0%** | 1,031 | **8.6%** | 1,420 | **11.9%** | 1,761 | **14.8%** | 2,126 | **17.8%** |
| 2010 | 12,160 | 454 | **3.7%** | 682 | **5.6%** | **40.0%** | 1,136 | **9.3%** | 1,493 | **12.3%** | 1,928 | **15.9%** | 2,266 | **18.6%** |
| 2011 | 11,774 | 755 | **6.4%** | 439 | **3.7%** | **63.2%** | 1,194 | **10.1%** | 1,536 | **13.0%** | 2,017 | **17.1%** | 2,345 | **19.9%** |
| 2012 | 12,224 | 886 | **7.2%** | 513 | **4.2%** | **48.6%** | 1,823 | **14.9%** | 1,741 | **14.2%** | 2,294 | **18.8%** | 2,643 | **21.6%** |
| 2013 | 12,321 | 874 | **7.1%** | 534 | **4.3%** | **62.1%** | 1,408 | **11.4%** | 1,846 | **15.0%** | 2,339 | **19.0%** | 2,693 | **21.9%** |
| 2014 | 11,795 | 952 | **8.1%** | 491 | **4.2%** | **49.9%** | 1,909 | **16.2%** | 1,925 | **16.3%** | 2,352 | **19.9%** | 2,750 | **23.3%** |
| 2015 | 10,348 | 898 | **8.7%** | 477 | **4.6%** | **48.4%** | 1,855 | **17.9%** | 2,014 | **19.5%** | 2,156 | **20.8%** | 2,561 | **24.7%** |
|  |  |  |  |  |  |  |  |  |  |  |  |  |  |  |
| All | 181,668 | 7,787 | **4.3%** | 7,062 | **3.9%** | **48.0%** | 16,219 | **8.9%** | 20,940 | **11.5%** | 24,649 | **13.6%** | 30,687 | **16.9%** |

* - Denominator is all deaths with any mention of dementia on death certificate
** - Denominator is all deaths with any mention of dementia on GP or HES record

**Supplementary Table 6:** Summary of dementia recording in GP and HES among all deaths certified with mention of dementia from 2001 to 2015 (n=19,627)

| **Year of Death** | **All deaths mentioning dementia** | **Has prior GP diagnosis** | | **Has prior HES diagnosis** | | **Has prior diagnosis (GP or HES)** | | **Has prior GP diagnosis other GP information suggestive of dementia** | | **Has prior diagnosis (GP or HES) or other GP information suggestive of dementia** | |
| --- | --- | --- | --- | --- | --- | --- | --- | --- | --- | --- | --- |
|  |  |  |  |  |  |  |  |  |  |  |  |
|  | **n** | **n** | **%** | **n** | **%** | **n** | **%** | **n** | **%** | **n** | **%** |
| 2001 | 733 | 389 | **53.1%** | 379 | **51.7%** | 559 | **76.3%** | 564 | **76.9%** | 566 | **77.2%** |
| 2002 | 820 | 429 | **52.3%** | 447 | **54.5%** | 618 | **75.4%** | 631 | **77.0%** | 634 | **77.3%** |
| 2003 | 919 | 520 | **56.6%** | 536 | **58.3%** | 747 | **81.3%** | 755 | **82.2%** | 758 | **82.5%** |
| 2004 | 927 | 543 | **58.6%** | 566 | **61.1%** | 774 | **83.5%** | 789 | **85.1%** | 791 | **85.3%** |
| 2005 | 968 | 622 | **64.3%** | 651 | **67.3%** | 831 | **85.8%** | 847 | **87.5%** | 849 | **87.7%** |
| 2006 | 1,014 | 627 | **61.8%** | 714 | **70.4%** | 887 | **87.5%** | 903 | **89.1%** | 906 | **89.3%** |
| 2007 | 1,087 | 738 | **67.9%** | 796 | **73.2%** | 975 | **89.7%** | 997 | **91.7%** | 999 | **91.9%** |
| 2008 | 1,287 | 885 | **68.8%** | 945 | **73.4%** | 1,146 | **89.0%** | 1,159 | **90.1%** | 1,170 | **90.9%** |
| 2009 | 1,374 | 927 | **67.5%** | 1,057 | **76.9%** | 1,238 | **90.1%** | 1,266 | **92.1%** | 1,273 | **92.6%** |
| 2010 | 1,478 | 950 | **64.3%** | 1,175 | **79.5%** | 1,337 | **90.5%** | 1,362 | **92.2%** | 1,366 | **92.4%** |
| 2011 | 1,565 | 1,052 | **67.2%** | 1,267 | **81.0%** | 1,447 | **92.5%** | 1,471 | **94.0%** | 1,475 | **94.2%** |
| 2012 | 1,823 | 1,252 | **68.7%** | 1,494 | **82.0%** | 1,694 | **92.9%** | 1,722 | **94.5%** | 1,730 | **94.9%** |
| 2013 | 1,868 | 1,295 | **69.3%** | 1,543 | **82.6%** | 1,753 | **93.8%** | 1,786 | **95.6%** | 1,792 | **95.9%** |
| 2014 | 1,909 | 1,355 | **71.0%** | 1,582 | **82.9%** | 1,797 | **94.1%** | 1,832 | **96.0%** | 1,836 | **96.2%** |
| 2015 | 1,855 | 1,487 | **80.2%** | 1,544 | **83.2%** | 1,776 | **95.7%** | 1,805 | **97.3%** | 1,813 | **97.7%** |
| All | 19,627 | 13,071 | **66.6%** | 14,696 | **74.9%** | 17,579 | **89.6%** | 17,889 | **91.1%** | 17,958 | **91.5%** |

**Supplementary Figure 1:** Summary of death recording by source in all deaths identified from CPRD database between 2001 and 2015

**153 practices** in CPRD recording data continuously 2001-2015

**207,068 (100%)**
Deaths identified on GP records & linked to ONS

**19,627 (9.5%)**
Dementia mentioned on ONS death certificate

**10,253 (5.0%)**
Dementia as underlying cause on ONS death certificate

**32,111 (15.5%)**
Dementia diagnosis previously on hospital record

**26,255 (12.7%)**
Dementia diagnosis previously on GP record

**Note:** A total of **1.7 million** patients were actively registered for any time during 2001-2015

**166,565 (80.4%)**
No Dementia recorded anywhere

**40,503 (19.6%)**
Dementia diagnosis in ANY of 3 sources

**Supplementary Figure 2:** Death rate for all forms of dementia, England and Wales 2001 to 2016 for males and females


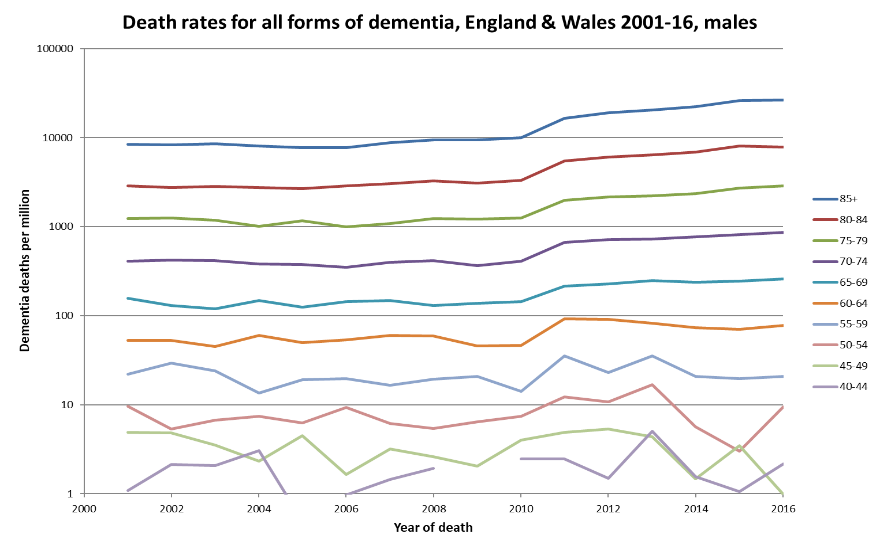

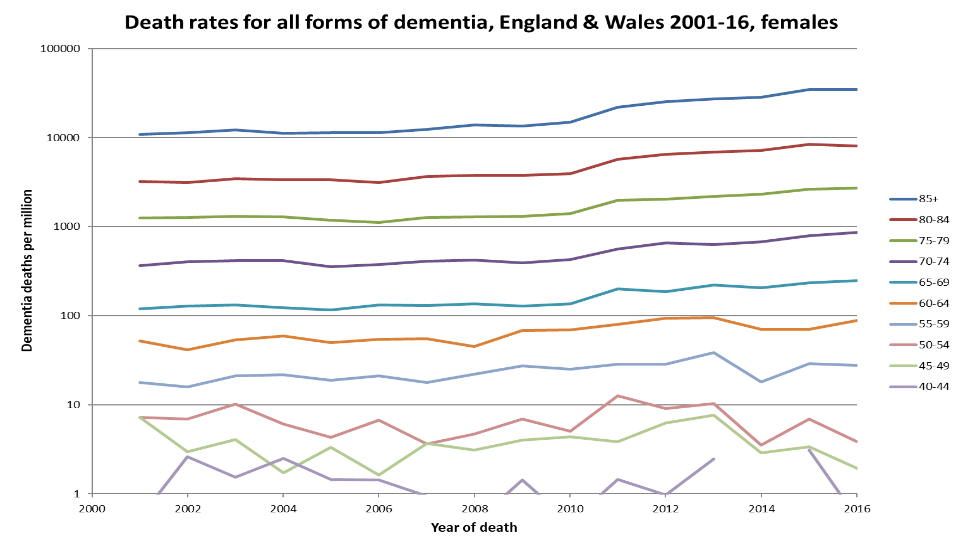


Footnotes:

1. These figures are based on data from the Office for National Statistics historical mortality datasets and have been plotted on a log scale

2. Restricted to ICD-10 (2001 onwards)

3. Coding changes occurred in 2001-02 (affecting vascular dementia mainly) and 2010-11 (affecting vascular and other specified forms of dementia much more than Alzheimer’s or unspecified)
